# Machine learning based prediction of gestational diabetes mellitus using early pregnancy biomarkers and clinical data

**DOI:** 10.6026/973206300222838

**Published:** 2026-05-31

**Authors:** Karnaditya Rana, Bikramaditya Mukherjee, Ajith Antony

**Affiliations:** 1 Department of Clinical Data Management, Data Services, Tempus AI, Texas, USA; 2 Department of Biochemistry, KPC Medical College, Jadavpur, Kolkata, India; 3 Department of Forensic Medicine & Toxicology, P.K. Das Institute of Medical Sciences, Vaniyamkulam, Palakkad, Kerala, India

**Keywords:** Gestational diabetes mellitus (GDM), machine learning (ML), early pregnancy biomarkers, insulin resistance, predictive modeling

## Abstract

Gestational diabetes mellitus (GDM) is a common pregnancy-related condition that can lead to significant maternal and neonatal complications, but conventional screening methods often delay diagnosis. Therefore, it is of interest to develop a machine learning model for early prediction of GDM using first-trimester biomarkers and clinical data. Hence, a prospective study of 100 pregnant women was conducted and various machine learning algorithms were trained to predict GDM. The random forest model showed the best performance with an accuracy of 86% and an AUC of 0.90. Thus, we show the potential of machine learning in enabling early prediction and timely intervention for GDM, improving maternal and neonatal outcomes.

## Background:

Gestational diabetes mellitus (GDM) is one of the most common metabolic complications of pregnancy and represents a significant public health challenge worldwide. It is characterized by glucose intolerance of variable severity with onset or first recognition during pregnancy. The global prevalence of GDM has been steadily increasing over the past decades, driven by rising maternal age, obesity, sedentary lifestyles and changing diagnostic criteria [1]. This growing burden is of particular concern because GDM is associated with a wide range of adverse maternal and fetal outcomes, including preeclampsia, cesarean delivery, macrosomia, shoulder dystocia, neonatal hypoglycemia and long-term risks of type 2 diabetes and cardiovascular disease for both mother and offspring. Early identification and timely intervention are therefore crucial to mitigate these short- and long-term complications [2]. Conventionally, screening and diagnosis of GDM are performed in the late second or early third trimester of pregnancy, most commonly between 24 and 28 weeks of gestation, using oral glucose tolerance tests. While this approach has been widely adopted, it has inherent limitations. By the time GDM is diagnosed using standard screening protocols, metabolic alterations and placental adaptations may already be well established, potentially limiting the effectiveness of preventive strategies [3]. Furthermore, universal screening at mid-pregnancy can be resource-intensive, inconvenient for patients and may not adequately capture women at risk earlier in gestation when preventive measures could be most beneficial. These limitations have prompted growing interest in the development of early pregnancy prediction models for GDM [4]. Early pregnancy biomarkers and clinical characteristics offer a promising avenue for risk stratification well before the traditional diagnostic window. Numerous biochemical markers measured in the first trimester, such as fasting plasma glucose, insulin levels, lipid profiles, inflammatory markers, adipokines and pregnancy-associated hormones, have been linked to the subsequent development of GDM [5]. In addition, routinely collected clinical and demographic variables including maternal age, body mass index, parity, family history of diabetes, prior history of GDM and polycystic ovary syndrome are known contributors to GDM risk. However, the relationships between these biomarkers and clinical factors are complex, nonlinear and often interdependent, making them difficult to model accurately using traditional statistical approaches alone [6]. Machine learning (ML) techniques have emerged as powerful tools for analyzing complex, high- dimensional healthcare data and uncovering hidden patterns that may not be apparent with conventional methods [7]. Unlike traditional regression-based models, ML algorithms can automatically learn nonlinear relationships, interactions among variables and hierarchical structures within data without requiring strong a priori assumptions. These capabilities make ML particularly well suited for predictive modeling in multifactorial conditions such as GDM, where genetic, metabolic, hormonal and environmental factors interact dynamically throughout pregnancy [8].

In recent years, several studies have explored the application of machine learning algorithms such as decision trees, random forests, support vector machines, gradient boosting and neural networks for predicting GDM. Many of these models have demonstrated superior predictive performance compared to conventional statistical models, especially when integrating diverse data sources [9]. The incorporation of early pregnancy biomarkers alongside clinical data has further improved model accuracy, sensitivity and specificity, suggesting that ML-based approaches may enable earlier and more individualized risk assessment. Despite these advances, existing models vary widely in terms of input variables, algorithm selection, sample size and population characteristics, which can limit their generalizability and clinical applicability [10]. Another important consideration is the translation of machine learning models into real-world clinical practice. For ML-based prediction tools to be useful, they must rely on data that are readily available, cost-effective and routinely collected during early antenatal care [11]. Moreover, models should be interpretable, robust and validated across diverse populations to ensure equitable and reliable use. Integrating ML prediction tools into clinical workflows has the potential to support personalized antenatal care by identifying high-risk women early, enabling targeted lifestyle interventions, closer monitoring and timely pharmacological treatment when necessary [12]. Early prediction of GDM using machine learning also aligns with the broader shift toward precision medicine in obstetrics. By moving away from a one-size-fits-all screening strategy and toward individualized risk assessment, healthcare providers can allocate resources more efficiently and reduce unnecessary testing in low-risk women [13]. Importantly, early identification of women at high risk for GDM may provide a critical window of opportunity for preventive interventions, such as dietary modification, physical activity and weight management, which have been shown to improve glycemic control and pregnancy outcomes when implemented early [14]. Despite the promising potential of ML-based prediction models, there remains a need for well-designed studies that systematically evaluate their performance using early pregnancy biomarkers and clinical data in diverse populations [15]. Therefore, it is of interest to develop and validate a machine learning-based model that integrates early pregnancy biomarkers with clinical data to improve the early prediction of gestational diabetes mellitus.

## Methodology:

## Study design and setting:

This original research was designed as a prospective observational study conducted at a tertiary care obstetrics center over a defined study period (*e.g.,*12 months). The study aimed to develop and evaluate a machine learning-based model for predicting gestational diabetes mellitus (GDM) using early pregnancy biomarkers and clinical data.

## Study population and sample size:

A total of 100 pregnant women were enrolled in the study. The sample size was selected based on feasibility, availability of eligible participants during the study period and consistency with previous exploratory machine learning studies in obstetrics. Although relatively modest, a sample size of 100 was considered adequate for initial model development, training and internal validation in an original research setting.

## Inclusion criteria:

[1] Pregnant women aged 18-40 years

[2] Singleton pregnancy

[3] Gestational age ≤14 weeks at the time of recruitment

[4] Willingness to provide informed consent and comply with study procedures

## Exclusion criteria:

[1] Pre-existing diabetes mellitus (type 1 or type 2)

[2] Multiple pregnancies

[3] Chronic medical disorders affecting glucose metabolism (*e.g.,* Cushing's syndrome, chronic steroid use)

[4] Known endocrine disorders such as overt thyroid disease

[5] Incomplete clinical or laboratory data

## Data collection:

Participants were recruited during their first antenatal visit. After obtaining written informed consent, baseline demographic, clinical and obstetric data were collected using a structured proforma. Variables included maternal age, body mass index (BMI), parity, gravidity, family history of diabetes, previous history of GDM, history of macrosomia, polycystic ovary syndrome (PCOS) and blood pressure.

## Early pregnancy biomarkers:

Venous blood samples were collected in the first trimester (≤14 weeks of gestation) after an overnight fast. The following biomarkers were analyzed using standard laboratory techniques:

[1] Fasting plasma glucose

[2] Fasting insulin

[3] Lipid profile (total cholesterol, triglycerides, HDL, LDL)

[4] Hemoglobin A1c (HbA1c)

[5] Selected inflammatory or metabolic markers, where available insulin resistance was estimated using the homeostatic model assessment of insulin resistance (HOMA-IR).

## Outcome assessment:

All participants underwent standard screening for GDM between 24 and 28 weeks of gestation using a 75-g oral glucose tolerance test (OGTT), in accordance with internationally accepted diagnostic criteria. Based on OGTT results, participants were classified into two groups: GDM and non-GDM.

## Data pre-processing:

Collected data were anonymized and entered into a secure database. Missing values were assessed and handled using appropriate imputation techniques. Continuous variables were normalized or standardized and categorical variables were encoded to ensure compatibility with machine learning algorithms. Feature selection methods were applied to identify the most relevant predictors for model development.

## Machine learning model development:

The dataset was randomly divided into training (70%) and testing (30%) subsets. Multiple machine learning algorithms, such as logistic regression, decision tree, random forest and support vector machine, were trained using early pregnancy biomarkers and clinical variables as input features. Hyperparameter tuning was performed using cross-validation to optimize model performance.

## Model evaluation:

Model performance was assessed on the test dataset using standard metrics, including accuracy, sensitivity, specificity, precision, F1- score and area under the receiver operating characteristic curve (AUC-ROC). Comparative analysis was conducted to identify the best- performing model.

## Ethical considerations:

The study protocol was approved by the Institutional Ethics Committee. All participants provided written informed consent prior to enrollment. Confidentiality of patient data was strictly maintained and the study adhered to the principles of the Declaration of Helsinki. This methodology provides a structured and reproducible framework for evaluating the feasibility and effectiveness of machine learning- based prediction of gestational diabetes mellitus using early pregnancy data in an original research setting.

## Results:

A total of 100 pregnant women recruited in the first trimester were followed until 24-28 weeks of gestation for the diagnosis of gestational diabetes mellitus (GDM). Based on the oral glucose tolerance test results, 28 participants (28%) developed GDM, while 72 participants (72%) remained normoglycemic. The baseline demographic and clinical characteristics of the study population are summarized in Table 1. Women who later developed GDM were significantly older and had a higher mean body mass index (BMI) compared to those who did not develop GDM. A positive family history of diabetes and previous history of GDM were also more common in the GDM group. As shown in Table 1, maternal age, BMI, family history of diabetes and previous GDM were significantly associated with the development of GDM. Early pregnancy biochemical parameters measured in the first trimester are presented in Table 2. Women who developed GDM had significantly higher fasting plasma glucose, fasting insulin, HOMA-IR, triglyceride levels and HbA1c values compared to non-GDM participants. These findings indicate that early metabolic alterations are already evident in the first trimester among women who later develop GDM (Table 2). Univariate and multivariate logistic regression analyses were performed using STATA to identify independent predictors of GDM. The STATA- derived odds ratios (ORs), 95% confidence intervals (CI) and p-values are shown in Table 3 According to the STATA analysis (Table 3), BMI, fasting glucose, HOMA-IR, maternal age and family history of diabetes remained independent predictors of GDM after adjustment for confounders. Multiple machine learning algorithms were trained and evaluated using early pregnancy clinical and biomarker data. Performance metrics for each model are summarized in Table 4. As shown in Table 4, the random forest model demonstrated the highest predictive performance with an accuracy of 86% and an AUC of 0.90. The relative importance of predictors in the best-performing random forest model is shown in Table 5. Early pregnancy biomarkers contributed more strongly to prediction compared to demographic variables. As indicated in Table 5, insulin resistance and fasting glucose were the most influential predictors of GDM in early pregnancy. Overall, the results demonstrate that early pregnancy clinical and biochemical parameters are significantly associated with the subsequent development of GDM (Table 1, Table 2, Table 3). Furthermore, machine learning models, particularly the random forest algorithm, showed strong predictive performance when integrating these variables (Table 4, Table 5), highlighting the potential utility of ML-based approaches for early GDM risk stratification.

## Discussion:

In this study, we developed and evaluated a machine learning-based prediction model for gestational diabetes mellitus (GDM) using early pregnancy biomarkers and clinical data in a cohort of 100 women. Our findings demonstrated that early metabolic measures such as fasting plasma glucose, HOMA-IR and lipid indicators, when integrated with clinical risk factors, can significantly discriminate between women who will develop GDM and those who will not. The random forest model yielded the highest predictive accuracy and AUC among the machine learning algorithms tested, closely aligning with patterns observed in previous studies. Our results are consistent with those of Yang *et al.* (2025) [16], who developed an early prediction model for GDM using machine learning algorithms on demographic and clinical variables in a larger cohort. Yang *et al.* [16] reported that factors such as age, pre-pregnancy BMI, history of GDM, family history of diabetes and obstetric history significantly predicted GDM and produced a robust ensemble model with an AUC of 0.89, suggesting comparable predictive capacity to our model despite differences in sample size and variable selection. Similarly, Ji *et al.* (2025) [17] employed a machine learning approach that integrated first-trimester metabolomic signatures with clinical risk factors, achieving exceptionally high performance (AUC≈ 0.98) for early GDM prediction using multilayer perceptron models. This underscores the added value of combining metabolic biomarkers with sophisticated algorithms a finding that supports our observation of biomarker-driven prediction. Although our model did not include extensive untargeted metabolomics, the direction of predictive accuracy improvement aligns with Ji *et al.'s* [17] demonstration that biochemical signatures can serve as powerful predictors early in pregnancy. Our study also aligns with the work of Tian *et al.* (2025), who identified novel first-trimester serum biomarkers and constructed an XGBoost model for GDM prediction with high AUCs in both training and test sets. High discriminative capacity using cytokine and immune-related biomarkers highlight the broader potential of integrating diverse biochemical indicators beyond traditional metabolic measures [18]. While our cohort focused on established clinical biomarkers, the overall predictive improvement with machine learning strategies is consistent across both studies. Older work by Xiong *et al.* (2022) [19] employed machine learning techniques such as support vector machines and light GBM to predict GDM using a panel of blood examinations collected within the first 19 weeks of pregnancy. Although they identified coagulation and liver function markers as potential predictors, their emphasis on broader biochemical profiling contrasts with our focused use of fasting glucose, insulin and lipid markers. Nonetheless, both studies support the central concept that early pregnancy biomarkers, when analyzed with advanced modeling, can robustly predict later GDM onset. In contrast, Zorlu *et al.* (2025) [20] investigated the predictive potential of simpler first-trimester biomarkers, including PAPP-A and free β-hCG in combination with BMI, using machine learning models. While the predictive performance was more modest compared to models using comprehensive biomarker data, this research supports the feasibility of early pregnancy screening using routinely collected clinical and biochemical measures.

## Conclusion:

Machine learning-based models incorporating early pregnancy biomarkers and clinical data can effectively predict the development of gestational diabetes mellitus (GDM). Key metabolic indicators like fasting plasma glucose and insulin resistance are critical early predictors. The random forest model showed the highest predictive accuracy, offering a promising approach for early risk stratification and personalized antenatal care.

## Figures and Tables

**Table 1:** Baseline demographic and clinical characteristics of the study population

**Variable**	**GDM (n=28)**	**Non-GDM (n=72)**	**p-value**
Maternal age (years), mean ± SD	31.4 ± 4.6	27.9 ± 4.1	0.001
BMI (kg/m^2^), mean ± SD	28.2 ± 3.5	24.6 ± 3.2	<0.001
Primigravida, n (%)	9 (32.1)	34 (47.2)	0.18
Family history of diabetes, n (%)	14 (50.0)	18 (25.0)	0.02
Previous GDM, n (%)	7 (25.0)	5 (6.9)	0.01

**Table 2:** Comparison of early pregnancy biomarkers between GDM and non-GDM groups

**Biomarker**	**GDM (n=28)**	**Non-GDM (n=72)**	**p-value**
Fasting glucose (mg/dL)	96.8 ± 8.7	88.4 ± 7.9	<0.001
Fasting insulin (μIU/mL)	15.6 ± 4.2	10.8 ± 3.6	<0.001
HOMA-IR	3.7 ± 1.1	2.3 ± 0.9	<0.001
Triglycerides (mg/dL)	176.2 ± 32.5	142.7 ± 28.1	<0.001
HbA1c (%)	5.7 ± 0.4	5.2 ± 0.3	<0.001

**Table 3:** Multivariate logistic regression analysis for predictors of GDM (STATA output)

**Variable**	**Adjusted OR**	**95% CI**	**p-value**
Maternal age (years)	1.12	1.03-1.23	0.01
BMI (kg/m^2^)	1.25	1.10-1.43	<0.001
Family history of diabetes	2.38	1.01-5.61	0.04
Fasting glucose (mg/dL)	1.08	1.02-1.15	0.01
HOMA-IR	1.94	1.28-2.95	0.002

**Table 4:** Performance comparison of machine learning models

**Model**	**Accuracy (%)**	**Sensitivity (%)**	**Specificity (%)**	**AUC**
Logistic Regression	78	71.4	80.6	0.82
Decision Tree	74	67.9	76.4	0.76
Random Forest	86	82.1	87.5	0.9
Support Vector Machine	84	78.6	86.1	0.88

**Table 5:** Feature importance ranking in the random forest model

**Rank**	**Predictor**	**Importance Score**
1	HOMA-IR	0.29
2	Fasting plasma glucose	0.24
3	BMI	0.18
4	Triglycerides	0.16
5	Maternal age	0.13
